# Investigation on Distribution and Risk Assessment of Volatile Organic Compounds in Surface Water, Sediment, and Soil in a Chemical Industrial Park and Adjacent Area

**DOI:** 10.3390/molecules26195988

**Published:** 2021-10-02

**Authors:** Rongrong Lei, Yamei Sun, Shuai Zhu, Tianqi Jia, Yunchen He, Jinglin Deng, Wenbin Liu

**Affiliations:** 1 Research Center for Eco-Environmental Sciences, Chinese Academy of Sciences, Beijing 100085, China; leirongr@163.com (R.L.); tqijia@126.com (T.J.); yunchenhe@yeah.net (Y.H.); dengjlin@yeah.net (J.D.); 2 University of Chinese Academy of Sciences, Beijing 100049, China; 3 Chinese Academy of Environmental Planning, Beijing 100012, China; 4 National Research Center for Geoanalysis, Beijing 100037, China; zhu15131215153@126.com; 5 Hangzhou Institute for Advanced Study, UCAS, Hangzhou 310024, China

**Keywords:** VOCs, distribution, environmental media, risk assessment, chemical industrial park

## Abstract

The occurrences, distributions, and risks of 55 target volatile organic compounds (VOCs) in water, sediment, sludge, and soil samples taken from a chemical industrial park and the adjacent area were investigated in this study. The Σ_55_-VOCs concentrations in the water, sediment, sludge, and soil samples were 1.22–5449.21 μg L^−1^, ND–52.20 ng g^−1^, 21.53 ng g^−1^, and ND–11.58 ng g^−1^, respectively. The main products in this park are medicines, pesticides, and novel materials. As for the species of VOCs, aromatic hydrocarbons were the dominant VOCs in the soil samples, whereas halogenated aliphatic hydrocarbons were the dominant VOCs in the water samples. The VOCs concentrations in water samples collected at different locations varied by 1–3 orders of magnitude, and the average concentration in river water inside the park was obviously higher than that in river water outside the park. However, the risk quotients for most of the VOCs indicated a low risk to the relevant, sensitive aquatic organisms in the river water. The average VOCs concentration in soil from the park was slightly higher than that from the adjacent area. This result showed that the chemical industrial park had a limited impact on the surrounding soil, while the use of pesticides, incomplete combustion of coal and biomass, and automobile exhaust emissions are all potential sources of the VOCs in the environmental soil. The results of this study could be used to evaluate the effects of VOCs emitted from chemical production and transportation in the park on the surrounding environment.

## 1. Introduction

Volatile organic compounds (VOCs) are a class of organic chemicals that easily vaporize and enter the environment at room temperature. There is great concern about the potential harm they can cause to human health and the environment [1,2,3,4,5]. VOCs are mainly emitted from biological and anthropogenic sources [6,7] and are widespread in the environment because of their volatility. Anthropogenic VOCs are important precursors in the production of ozone and secondary organic aerosols, which have a significant impact on the atmospheric environment [8,9,10]. VOCs are extensively released from fuels, paints, spray, solvents, deodorants, combustion exhausts, etc. [11]. They are released into the environment during their production, storage, handling, and use and can infiltrate surface water, soil, and sediments through various physical and chemical processes from many sources [12]. Many VOCs are classified as toxic and carcinogenic pollutants, and exposure to VOCs can increase the risk of illness, congenital malformation, neurocognitive impairment, and cancer in humans [11].

The emission of VOCs from anthropogenic sources is a major issue in China. In 2012, 29.85 million tons of VOCs were emitted in China, and the emissions from industry sources in 2018 totaled 12.7 million tons [13,14]. As a key source of VOCs in China, the chemical industry accounts for more than 40% of the total emissions from industrial sources nationally [15]. The chemical industry plays an important role in economic development. However, chemical industrial parks contain many plants that manufacture multiple products and generate associated, complex pollutants and emissions, which are difficult to monitor and regulate. As an important emission source of various pollutants, chemical industrial parks will have an adverse impact on the surrounding environment. Studies have shown that the concentrations and risks of pollutants, such as pesticides, pharmaceutical pollutants, polycyclic aromatic hydrocarbons, and some VOCs, are relatively high in various environmental media surrounding chemical industrial parks [16,17,18].

Previous studies on VOCs mainly focus on air samples and few on environmental media such as water and soil. To evaluate the effects of VOCs emissions from chemical production and transportation in chemical industrial parks on the surrounding environment, we comprehensively investigated the occurrences, distributions, and risks of 55 target VOCs in water, soil, and sediment samples from a selected park and an adjacent area. There are more than 100 chemical plants in the park, and the main products are medicines, pesticides, and novel materials. The analysis of the concentration levels and composition characteristics of the VOCs in the samples suggests a source-to-sink relationship, with the chemical industrial park as the main emission source. The impact of production and transportation in the chemical park on the surrounding environment was studied, and the ecological environmental risk of VOCs was evaluated. The results will contribute to the knowledge of potential influences of VOCs from chemical industrial parks on the environment, and these are also helpful for risk management and the control of pollutants in chemical industrial parks.

## 2. Materials and Methods

### 2.1. Chemicals and Reagents

A total of 55 compounds (Table S1, in the Supplementary Materials) were monitored. VOC mixed standards, surrogate standards (fluorobenzene and 4-bromofluorobenzene), and internal standards (dibromofluoromethane, toluene-*d*_8_, and 1,4-dichlorobenzene-*d*_4_) referenced to US EPA Method 8260D [19] were purchased from o2si Smart Solutions (North Charleston, SC, USA). Methanol and dichloromethane were pesticide residue grade and were purchased from J.T. Baker Chemical Company (Phillipsburg, NJ, USA).

### 2.2. Sampling

**Figure 1:**
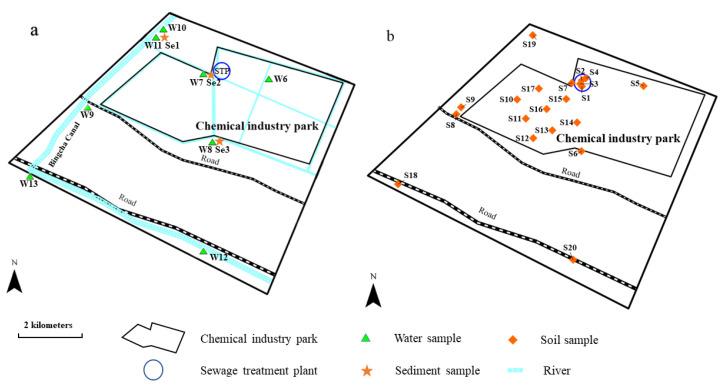
Map of the sampling sites. (**a**: water and sediment samples, **b**: soil samples).

Rudong Yangkou Chemical Industrial Park has an area of 11.6 km^2^ and is located in Nantong, Jiangsu Province, China. In November 2018, we collected a series of water, soil, and sediment samples in the park and the surrounding area. A map of the sampling sites is presented in Figure 1.

The differences between the VOCs concentrations in samples collected inside and outside of the park could reflect the influence of the production activities in the chemical industrial park on the nearby environment. To compare the VOCs concentrations and compositions, different samples collected inside and outside the chemical industrial park were analyzed. Five sewage samples (labeled W1–W5) were collected from the sewage treatment plant (STP) at the water inlet (W3–W5), the regulating tank (W1), and the water outlet (W2). One sludge sample was collected from the STP. Eight surface water samples (labeled W6–W13) were collected from the river water inside the chemical industrial park (InCP) and the river water outside the chemical industrial park (OuCP). Because the river bank and bottom were sealed with cement, only three sediment samples (labeled Se1–Se3) were collected from a depth of 0–20 cm at different sites in the rivers using a stainless-steel grab sampler. All the water samples were collected in 40 mL pre-cleaned brown glass sampling bottles. The sediment and sludge samples were stored in 60 mL screw-top wide-mouth jars.

Twenty surface soil samples (labeled S1–S20) were collected in the park and the adjacent area from a depth of 0–20 cm using a stainless-steel scoop (Figure 1b). Each soil sample was a composite of five samples collected from the four corners and the center of a square (5 m × 5 m) at each sampling site. A portion of the mixed sample was used to fill a 60 mL screw-top wide-mouth jar, and the jar was rapidly sealed and retained for VOC analysis.

To prevent effusion and photolysis of the analytes, all the samples were collected into brown glass sampling bottles that were filled completely without headspace. The samples were stored in the dark at 4 °C in a portable refrigerator and analyzed within 48 h.

### 2.3. Sample Analysis

The VOCs were analyzed using a gas chromatograph/mass spectrometer (GC/MS) (Agilent 7890B/5977A, Agilent Technologies Inc., Santa Clara, CA, USA) that was equipped with a purge and trap (P&T) concentrator (Tekmar-3100, Tekmar Dohrmann, Mason, OH, USA). The sample analysis followed EPA Method 8260D [19]. The water samples were directly analyzed, but the soil and sediment samples were pre-treated before analysis. First, approximately 5 g of the sample was accurately weighed into a 40 mL brown bottle, and then 1 g of sodium bisulfate, 5 mL of ultrapure water, and a magnetic stirring bar were added. Surrogate and internal standards (1 μL, 30 mg L^−1^) were added to the sample by the autosampler during analysis.

The parameters of the P&T were set as follows: sample volume, 5 mL; purge gas, helium (99.999%); flow rate, 40 mL min^−1^; purge time, 11 min; purge temperature, 20 °C; desorption temperature, 190 °C; desorption time, 2 min; baking temperature, 210 °C; and hold time, 10 min.

A capillary column (Agilent DB-624, 30 m × 0.25 mm, 1.4 μm) was used for the GC/MS. The injection temperature was 190 °C, and the split ratio was 20:1. Helium (99.99%) was used as the carrier gas at a flow rate of 1.73 mL min^−1^. The oven temperature was held at 45 °C for 2 min, increased to 120 °C at 6 °C min^−1^, and then increased to 220 °C at 12 °C min^−1^ and held for 5 min. Electron ionization was performed at 70 eV with an ion source temperature of 200 °C. The temperature of the quadrupole rods was 150 °C, and the interface temperature was 210 °C. The MS was operated in scan mode (45.00–280.00 amu).

### 2.4. Quality Assurance and Quality Control

Strict quality assurance and quality control measures were performed for field sampling and laboratory analyses. The recovery for surrogates was maintained in the range of 72–118%. The concentrations of target compounds in laboratory blanks and procedural blanks were below the method detection limits. The recovery range of spiked sample matrices was 83–114%.

### 2.5. Environmental Risk Assessment

The environmental risks assessment of the VOCs in the water to the aquatic organisms followed the method used in previous research [5,20]. The risk quotient (RQ) model can be used to quantify the risk of exposure of a specific species to the chemicals in the surrounding natural environment [4]. The measured environmental concentration (MEC, μg L^−1^) of an individual compound and the predicted no-effect concentration (PNEC, μg L^−1^) to organisms are used to calculate the RQ as follows:

(1) $$\begin{array}{c} RQ_{i}=\frac{MEC_{i}}{PNEC_{i}} \end{array}$$

PNECs are derived from toxicity test data (acute and chronic toxicity, such as lethal concentration (LC_50_), effective concentration (EC_50_), and chronic value (ChV) and the assessment factor (AF). In this study, the minimum ChV of the individual chemicals for fish, *Daphnia*, and green algae were used to calculate the PNECs. As the toxicity data of three trophic levels were selected here, the AF was set to 10, according to the literature (Van Leeuwen and Vermeire, 2007). The PNECs were calculated using the following equation:

(2) $$\begin{array}{c} PNEC_{i}\equiv \frac{min(ChV_{i,fish},\;ChV_{i,\;Daphnia},\;ChV_{i,\;green\;algea})}{10} \end{array}$$

The ChVs (μg L^−1^) were obtained using ECOSAR V2.0 software [21], and the relevant data are given in Table S1 (in the Supplementary Materials). The sum of the RQ (RQ_total_) for all the detected compounds was calculated to estimate the total toxicity using the following equation:

(3) $$\begin{array}{c} RQ_{total}=RQ_{1}+RQ_{2}+\cdots +RQ_{i} \end{array}$$

## 3. Results and Discussion

### 3.1. VOCs in Water, Sediment, and Sludge

The Σ_55_-VOCs concentrations in the water, sediment, and sludge samples collected from different sites within and around the chemical industrial park were analyzed for detection rates, total concentrations, and compositions of VOCs (Table 1). The detection rate of VOC species in the water samples (2–74%) extended to a higher value than in the sludge (19%) or sediment (0–22%) samples. The VOCs concentrations in the water samples from different locations varied by 1–3 orders of magnitude. The VOCs concentrations in the rivers from the study area were obviously higher than those in other environmental rivers in China, such as Dongjiang Lake (2.93 to 4.69 µg/L) [3], Yangtze River (0–4.03 µg/L), Huaihe River (0–22.21 µg/L), Yellow River (1.94–42.78 µg/L), Haihe River (0.48–42.78 µg/L), and Liaohe River (1.10–20.85 µg/L) [4].

The VOCs concentrations at some points in the chemical park are very high, which may be caused by unorganized emissions or leakage in the process of chemical production and transportation. Therefore, the unorganized discharge of VOCs from some enterprises in the parks should be controlled. Meanwhile, measures should be taken to minimize the leakage of VOCs during transportation.

#### 3.1.1. Samples from the Sewage Treatment Plant

The STP is a sink of pollutants in the chemical industrial park, and the treatment efficiency of pollutants in the STP is important in regulating the impact of pollutants on the surrounding environment. Water samples taken from the inlet, the regulating tank, and the outlet of the STP were analyzed in this study. Sewage discharged by specific enterprises in the inlet of the STP represented the VOCs concentrations and emission characteristics of these enterprises. Samples W3–W5 were collected from sewage originating from enterprises that produce polymer materials and drug intermediates; agricultural chemicals; and pesticide intermediates and formulations, respectively. The VOCs compositions in these samples were different. Samples W3 and W4 were dominated by alkenyl halides (75%) and alkyl halides (93%), respectively. The dominant components in sample W5 were alkenyl halides (50%) as well as aryl halides and aromatic hydrocarbons (50%). The differences in the VOCs compositions in these samples could be used as a reference for source analysis of the VOCs in the environment.

**Figure 2:**
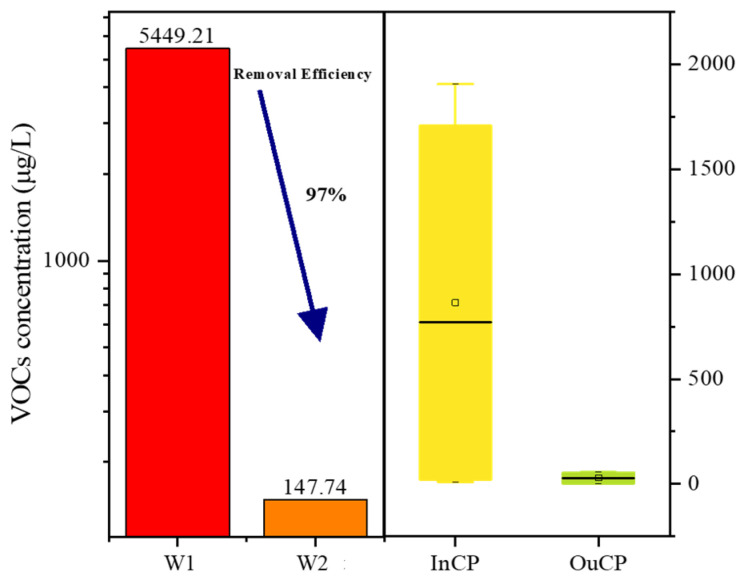
VOCs concentrations in the water samples collected at the sewage treatment plant (W1, W2), inside the park (InCP), and outside the park (OuCP).

The sample taken from the regulating tank represents the overall level and characteristics of the VOCs in the chemical industrial park. The difference in the VOCs concentrations between the regulating tank (W1) and the outlet (W2) reflects the removal efficiency of the VOCs in the STP (Figure 2). As expected, W1 contained the most VOCs (40 species) at the highest concentrations (up to 5449.21 μg L^−1^). The dominant compound in the sample from the regulating tank was dichloromethane (87%), and the total contribution of alkyl halides and alkenyl halides was up to 99% of all compounds in the sample. These results could be attributed to the fact that the main products manufactured in the studied chemical industrial park are medicines, pesticides, and novel materials, and alkyl halides and alkenyl halides are important compounds used in the synthesis of drugs and polymer materials. Compared with the regulating tank sample, the sample from the effluent outlet of the STP had a much lower concentration (147.74 μg L^−1^) and a variety of VOCs. According to our results, the VOC-removal rate of the STP was up to approximately 97%. However, although most of the VOCs were reduced in concentration or removed from the sewage after treatment, the concentration of benzene significantly increased. This could be attributed to the fact that most aromatic hydrocarbons and aryl halides are degraded and converted into benzene during sewage treatment, but they are not completely decomposed and removed. Ten different VOCs were detected in the sludge from the STP, and the total VOCs concentration was 21.54 ng g^−1^. These results imply that VOCs adsorbed in the sludge are degraded during sewage treatment.

#### 3.1.2. Comparison of VOCs in River Water Inside and Outside the Chemical Industrial Park

The average VOCs concentrations in the InCP samples were obviously higher than those in the OuCP samples (Figure 2). However, for individual samples, the VOCs concentrations in some InCP samples were lower than those in some of the OuCP samples. Among the OuCP samples, the highest VOCs concentrations were found in the water from the Bingcha Canal (site W13), which is located about 3 km from the chemical industrial park. Moreover, the sample taken from this waterway also contained more VOC species than any of the other samples, except for the sample from the STP regulating tank. The high VOCs concentrations and number of species in this waterway may have been caused by the intensive use of river transportation and human activities.

**Figure 3:**
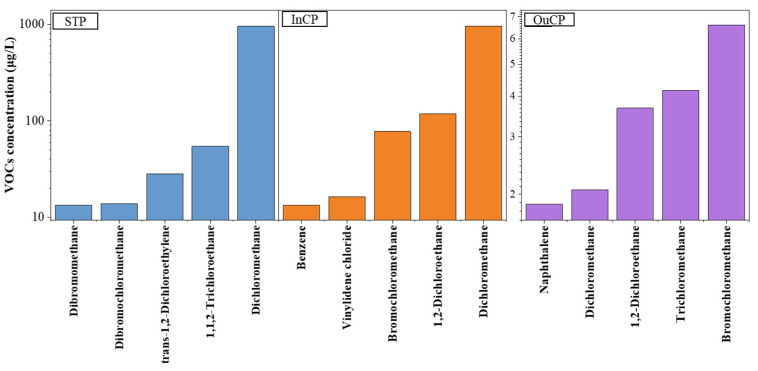
The top five VOC species in water samples collected at the sewage treatment plant (STP), inside the park (InCP), and outside the park (OuCP).

The top five abundant VOC species in the different types of samples are shown in Figure 3. The most abundant VOC species was dichloromethane in the samples from the STP and the InCP and bromochloromethane in the samples from the OuCP. The results were different from the other research where chloroform was the compound with the highest concentration in the surface water from a chemical industrial park [22]. This indicated that the VOCs released by the chemical industrial processes directly affected the environment. However, chemical industrial park’s industrial processes are not the only source of VOCs for samples collected outside a chemical industrial park. Benzene, toluene, ethylbenzene, and xylene (BTEX) have received much attention due to frequent detection in the environment, with the widespread use of petroleum products as their main source [22,23]. The BTEX concentration ranges in the InCP and the OuCP samples in the present study were ND–3.97 μg L^−1^ (mean: 1.50 μg L^−1^) and 1.00–3.55 μg L^−1^ (mean: 1.91 μg L^−1^), respectively. Unlike the Σ_55_-VOCs, the BETX concentration in the OuCP samples was slightly higher than that in the InCP samples. This is because the production and the transportation of chemical products in the chemical industrial park are the only sources of BTEX for the InCP samples. In the case of the OuCP samples, the BTEX concentration is affected by a combination of chemical production, coal and biomass burning, automobile exhaust emissions, and other industrial activities such as iron and steel production [1].

### 3.2. VOCs in Soil

**Figure 4:**
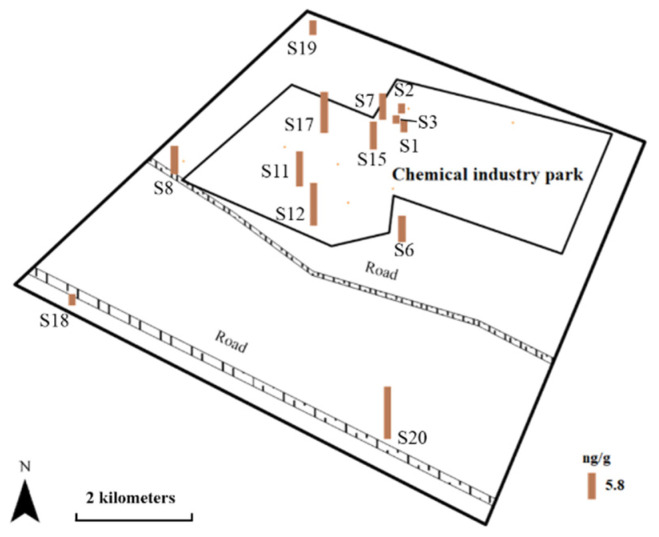
The distribution of total VOCs concentrations in soil at the study area.

Soil samples were collected from different sites, including inside the chemical industrial park, around the STP, and outside the park. The Σ_55_-VOCs concentration range in the soil samples was ND–11.58 ng g^−1^, which was lower than that in other chemical industrial parks [22]. The distribution of total VOCs concentrations in the soil at the study site is shown in Figure 4. The average VOCs concentration in the soil from inside the park was slightly higher than that in the soil from outside the park, but the VOCs were not detected in some samples. The Σ_55_-VOCs concentration in the soil from inside the park had a wide range (ND–9.16 ng g^−1^), and in the samples where VOCs were detected, the concentrations were generally higher, and more species were found than in the soil from outside the park. These results implied that the chemical production processes affected the concentrations and compositions of VOCs in the soil inside the chemical park. The highest Σ_55_-VOCs concentration was found in a sample (S20) from outside the park (11.58 ng g^−1^), which was collected in a small vegetable garden near the road. The largest contributor to the Σ_55_-VOCs concentration in this sample was *tert*-butylbenzene, which accounted for 41% of the total VOCs. *tert*-Butylbenzene is an important intermediate in the production of pharmaceuticals and pesticides, such as fungicides and acaricides [24]. This result suggests that the use of pesticides could be a major contributor to VOCs in agricultural soil.

Five kinds of VOCs were detected in the soil samples. These were 1,2,4-trimethylbenzene, benzene, *p*-isopropyl benzene, *tert*-butylbenzene, and *sec*-butylbenzene, which are all aromatic hydrocarbons. This was a little different from what was detected at the chemical industrial park in eastern China, which had more alkyl halide in the soil in the park [22]. In this study, benzene was the most frequently detected compound in the soil samples. These results were completely different from those obtained from the water samples, where the main VOCs were halogenated aliphatic hydrocarbons. This difference can be explained by the physical and chemical properties of the VOCs. Compared with aromatic hydrocarbons, aliphatic hydrocarbons have higher water solubility and lower octanol–water distribution coefficients, which lead to different distribution characteristics in water and soil. The concentrations of BTEX in all soil samples ranged from ND to 3.52 ng g^−1^ (mean: 1.55 ng g^−1^), which was close to the relatively low levels of BTEX in water. Generally, the chemical industrial park had a limited impact on the surrounding soil. The potential sources of VOCs in the soil in the surrounding environment are the use of pesticides, the incomplete combustion of coal and biomass, and automobile exhaust emissions [25,26].

### 3.3. Environment Risk Assessment

The RQs to fish, *Daphnia*, and green algae for individual VOCs in the InCP and the OuCP water samples were analyzed in this study. An RQ > 1.0 indicates high risk, an RQ of 0.1–1 indicates moderate risk, and an RQ of 0.01–0.1 indicates low risk [3]. The RQs for most of the VOCs, except for hexachlorobutadiene (HCBD), dichloromethane, 1,2-dichloroethane, vinylidene chloride, 1,2,3-trichlorobenzene, *n*-butylbenzene, and naphthalene, detected in eight sampling locations from the InCP and the OuCP were below 0.1. Therefore, these compounds were of low risk to the relevant, sensitive aquatic organisms in the area. Among the sampling locations, W13 (from the OuCP) had the highest RQ, which was 3.32 for HCBD. This result indicated that aquatic organisms were at potentially high risk from HCBD in this waterway. HCBD mainly comes from the production of chlorinated hydrocarbons, paints, plastics, and herbicides [27]. W13 is in an important transportation location, and the potentially high risk of HCBD may be caused by the volatilization and leakage of chemicals during transportation. Therefore, the management of the transportation and storage of chemicals outside the park should be strengthened.

High concentrations of dichloromethane at sampling locations W6 and W8 (from the OuCP) resulted in RQs for dichloromethane that were greater than one. Therefore, dichloromethane is of concern at these sites. The environmental risk of a pollutant is affected not only by its concentration in the environment but also by the pollutant toxicity and the organism sensitivity. Consequently, high pollutant concentrations are not directly related to environmental risk, even though they may induce adverse effects or represent an ecological risk [4,5]. In the present study, dichloromethane did not have the highest RQ, even though it was present in many samples and had the highest concentration among the VOCs in some samples. By contrast, the concentration of hexachlorobutadiene in sample W13 was far lower than that of dichloromethane in samples W6 and W8, but hexachlorobutadiene showed a higher RQ than dichloromethane for the selected aquatic organisms. Furthermore, the RQs for 1,2-dichloroethane, vinylidene chloride, 1,2,3-trichlorobenzene, *n*-butylbenzene, and naphthalene at some locations were higher than 0.1, which indicated that these compounds may pose a moderate risk to aquatic organisms.

The RQ_total_ values at the eight locations varied from 3.10 × 10^−3^ (sample W11) to 4.36 (sample W13). The RQ values for all the detected VOCs in the samples are shown in Table 2. Among the locations, the OuCP water sample collected in the Bingcha Canal had the highest RQ. As mentioned above, the VOCs concentration in this river was higher than that in the water taken from the other OuCP locations. Therefore, the future treatment of water in this river should focus on the removal of VOCs.

## 4. Conclusions

Chemical production had a great impact on the environment inside the chemical industrial park, and the VOCs concentrations of samples taken from inside the park were higher. It is found that the sewage treatment plant has high removal efficiency of VOCs in sewage through the study of the VOCs concentrations and species in the inlet and the outlet of the sewage treatment plant. However, the concentration of the VOCs in the sludge from the sewage treatment plant was higher than that in the soil and the sediment; therefore, careful disposal of the sludge should be considered. Based on the results from the soil and sediment samples, the chemical industrial park had a limited impact on the soil in the surrounding area.

One of the most important measures to control the effect of VOCs in the environment is to control the source emissions of VOCs. In this study, we found that chemical production has an important effect on the distribution of VOCs inside the chemical industrial park, but the emission sources of VOCs in the actual environment are complex. Pesticides, the incomplete combustion of coal and biomass, and automobile exhaust emissions are all potential sources of VOCs in the environment. Therefore, while paying attention to fixed emission sources, such as chemical industrial parks, these scattered, mobile emission sources should also be examined further.

## Figures and Tables

**Table 1:** VOCs concentrations and compositions in different samples from sewage treatment plant (STP), inside the park (InCP), and outside the park (OuCP).

Sample No.	Location	Count of Detected VOCs	Concentration (μg L^−1^ or ng g^−1^)	Composition			
				Alkyl Halide	Alkenyl Halide	Aryl Halide	Aromatic Hydrocarbon
W1	STP	40	5449.21	97.5%	1.6%	0.6%	0.3%
W2	STP	19	147.74	61.8%	6.0%	4.3%	27.9%
W3	STP	10	88.86	16.3%	74.9%	4.2%	4.6%
W4	STP	5	36.19	92.9%	7.1%	/	/
W5	STP	4	19.08	/	49.9%	26.1%	24.0%
W6	InCP	8	1905.99	99.5%	0.1%	0.1%	0.3%
W7	InCP	4	10.23	91.7%	/	/	8.3%
W8	InCP	6	1512.09	99.7%	0.2%	/	0.1%
W9	InCP	3	30.19	100.0%	/	/	/
W10	OuCP	5	54.36	96.6%	/	/	3.4%
W11	OuCP	1	1.22	/	/	/	100.0%
W12	OuCP	3	3.45	71.0%	/	/	29.0%
W13	OuCP	20	57.07	31.7%	9.8%	22.1%	36.4%
Slu *	STP	10	21.53	32.1%	30.0%	/	37.8%
Se1	OuCP	0	ND	/	/	/	/
Se2	InCP	1	1.09	/	/	/	100.0%
Se3	InCP	12	52.20	4.9%	44.8%	6.4%	43.9%
S1	STP	1	2.54	/	/	/	100.0%
S2	STP	1	2.00	/	/	/	100.0%
S3	STP	1	1.75	/	/	/	100.0%
S4	STP	0	ND	/	/	/	/
S5	InCP	0	ND	/	/	/	/
S6	InCP	3	5.62	/	/	/	100.0%
S7	InCP	4	5.69	/	/	/	100.0%
S8	InCP	3	6.15	/	/	/	100.0%
S9	InCP	0	ND	/	/	/	/
S10	InCP	0	ND	/	/	/	/
S11	InCP	3	7.70	/	/	/	100.0%
S12	InCP	5	9.24	/	/	/	100.0%
S13	InCP	0	ND	/	/	/	/
S14	InCP	0	ND	/	/	/	/
S15	InCP	5	6.00	/	/	/	100.0%
S16	InCP	0	ND	/	/	/	/
S17	InCP	5	9.16	/	/	/	100.0%
S18	OuCP	1	2.43	/	/	/	100.0%
S19	OuCP	1	3.09	/	/	/	100.0%
S20	OuCP	4	11.58	/	/	/	100.0%

ND: not detected. * Slu is sludge sample from STP.

**Table 2:** RQs of water in the rivers from study area.

Location	Sample Site		RQ	
		Min	Max	Sum
InCP	W6	2.02 × 10^−3^	1.570	1.881
	W7	1.23 × 10^−3^	0.007	0.013
	W8	3.31 × 10^−3^	1.142	1.653
	W9	1.53 × 10^−3^	0.019	0.034
OuCP	W10	5.19 × 10^−3^	0.029	0.081
	W11	3.40 × 10^−3^	0.003	0.003
	W12	7.92 × 10^−4^	0.003	0.005
	W13	4.39 × 10^−3^	3.318	4.359

## Data Availability

Not applicable.

## Supplementary Materials

The following are available online: Table S1: PNEC values calculated using chronic values (ChVs) from ECOSAR.
