# Supplementary material for: Investigation on Distribution and Risk Assessment of Volatile Organic Compounds in Surface Water, Sediment, and Soil in a Chemical Industrial Park and Adjacent Area
Source: Molecules. 2021 Oct 2;26(19):5988. doi: 10.3390/molecules26195988 (PMC8512396; doi:10.3390/molecules26195988)
Supplement: Supplementary file 1 [file molecules-26-05988-s001.zip › molecules-1367623-supplementary.pdf]

# Investigation on Distribution and Risk Assessment of Volatile Organic Compounds in Surface Water, Sediment, and Soil in a Chemical Industry Park and Adjacent Area

Rongrong Lei [1,4], Yamei Sun [2]*, Shuai Zhu [3], Tianqi Jia [1,4], Jinglin Deng [1,5], Yunchen He [1,4], Wenbin Liu [1,4,5]*

[1] Research Center for Eco-Environmental Sciences, Chinese Academy of Sciences, Beijing 100085, China

[2] Chinese Academy of Environmental Planning, Beijing, 100012, China

[3] National Research Center for Geoanalysis, Beijing,100037, China

[4] University of Chinese Academy of Sciences, Beijing 100049, China.

[5] Hangzhou Institute for Advanced Study, UCAS, Hangzhou 310024, China

*Correspondence: sunym@caep.org.cn (Y. Sun) liuwb@rcees.ac.cn (W. Liu). Tel.: +86-10-62849356; Fax: +86-10-62923563

## Contact Information:

**Corresponding author.**

*E-mail address*: sunym@caep.org.cn (Y. Sun) liuwb@rcees.ac.cn (W. Liu).

Table S1 PNEC values calculated using chronic values (ChVs) from ECOSAR

| **Compound** | **CAS** | **Limit of Detection** | | **ChV (µg/L)** | | | **PNEC (µg/L)** |
|---|---|---|---|---|---|---|---|
| | | **water µg/L** | **Soil ng/g** | **Fish** | ***Daphnid*** | **Green Algae** | |
| 1,1,1,2-Tetrachloroethane | 630-20-6 | 0.40 | 0.40 | 2166 | 1536 | 4181 | **154** |
| 1,1,1-trichloroethane | 71-55-6 | 0.20 | 0.80 | 2830 | 1890 | 4730 | **189** |
| 1,1,2,2-Tetrachloroethane | 79-34-5 | 0.10 | 0.50 | 9242 | 5484 | 11690 | **548** |
| 1,1,2-trichloroethane | 79-00-5 | 0.30 | 1.00 | 10461 | 5943 | 11937 | **594** |
| 1,1-Dichloropropane | 78-99-9 | 0.40 | 0.40 | 5568 | 3349 | 7274 | **335** |
| 1,1-dichloroethane | 75-34-3 | 0.10 | 0.50 | 12800 | 6820 | 12600 | **682** |
| 1,2,3-Trichloropropane | 96-18-4 | 0.40 | 0.40 | 4417 | 2824 | 6671 | **282** |
| 1,2-Dichloropropane | 78-87-5 | 0.20 | 1.00 | 5568 | 3349 | 7274 | **335** |
| 1,2-dichloroethane | 107-06-2 | 0.30 | 0.80 | 11052 | 6012 | 11376 | **601** |
| 1,2-Dibromo-3-chloropropane | 96-12-8 | 0.40 | 0.40 | 4990 | 3330 | 8340 | **333** |
| 1,2-Dibromoethane | 106-93-4 | 0.40 | 0.40 | 14775 | 8391 | 16844 | **839** |
| 1,3-Dichloropropane | 142-28-9 | 0.40 | 0.40 | 4821 | 2952 | 6569 | **295** |
| 2,2-Dichloropropane | 594-20-7 | 0.40 | 0.40 | 1507 | 1064 | 2881 | **106** |
| Dichloromethane | 75-09-2 | 0.50 | 1.20 | 24800 | 12000 | 19300 | **1200** |
| Dibromomethane | 74-95-3 | 0.40 | 0.40 | 35781 | 18059 | 30820 | **1806** |
| Dibromochloromethane | 124-48-1 | 0.20 | 1.00 | 30101 | 15866 | 28742 | **1587** |
| Trichloromethane | 67-66-3 | 0.20 | 0.80 | 24500 | 12369 | 21121 | **1237** |
| carbon tetrachloride | 56-23-5 | 0.20 | 0.80 | 5195 | 3273 | 7577 | **327** |
| Bromodichloromethane | 75-27-4 | 0.20 | 1.00 | 28215 | 14555 | 25599 | **1456** |
| Tribromomethane | 75-25-2 | 0.50 | 1.00 | 30652 | 16507 | 30801 | **1651** |
| Bromochloromethane | 74-97-5 | 0.40 | 0.40 | 31735 | 15676 | 25974 | **1568** |
| Vinylidene chloride | 75-35-4 | 0.40 | 0.80 | 5860 | 86 | 4154 | **8.6** |
| trans-1,2-Dichloroethylene | 156-60-5 | 0.20 | 0.80 | 8514 | 95 | 4942 | **9.5** |
| Trans-1,3-dichloropropene | 10061-02-6 | 0.10 | 0.50 | 4140 | 86 | 3800 | **8.6** |
| Hexachlorobutadiene | 87-68-3 | 0.40 | 0.40 | 12 | 33 | 392 | **1.2** |
| Vinyl chloride | 75-01-4 | 0.50 | 1.20 | 14800 | 80 | 5050 | **8.0** |
| Trichloroethylene | 79-01-6 | 0.20 | 1.00 | 2950 | 89 | 3550 | **8.9** |
| cis-1,2-Dichloroethene | 156-59-2 | 0.20 | 0.80 | 8514 | 95 | 4942 | **9.5** |
| cis-1,3-dichloropropene | 10061-01-5 | 0.10 | 0.50 | 4139 | 86 | 3799 | **8.6** |
| Perchloroethylene | 127-18-4 | 0.20 | 1.00 | 951 | 78 | 2378 | **7.8** |
| Chloroprene | 126-99-8 | 0.50 | 1.00 | 1720 | 58 | 2238 | **5.8** |
| 1,2,3-Trichlorobenzene | 87-61-6 | 0.40 | 0.40 | 334 | 301 | 1138 | **30** |
| 1,2,4-Trichlorobenzene | 120-82-1 | 0.20 | 0.80 | 334 | 301 | 1138 | **30** |

| | | | | | | | |
|---|---|---|---|---|---|---|---|
| 1,2-dichlorobenzene | 95-50-1 | 0.10 | 0.80 | 958 | 738 | 2256 | **74** |
| 1,3-dichlorobenzene | 541-73-1 | 0.10 | 0.80 | 958 | 738 | 2256 | **74** |
| 1,4-dichlorobenzene | 106-46-7 | 0.10 | 0.80 | 958 | 738 | 2256 | **74** |
| O-Chlorotoluene | 95-49-8 | 0.40 | 0.40 | 998 | 752 | 2224 | **75** |
| p-chlorotoluene | 106-43-4 | 0.40 | 0.40 | 998 | 752 | 2224 | **75** |
| Chlorobenzene | 108-90-7 | 0.10 | 0.80 | 2592 | 1712 | 4227 | **171** |
| Bromobenzene | 108-86-1 | 0.40 | 0.40 | 2235 | 1566 | 4193 | **157** |
| 1,2,4-Trimethylbenzene | 95-63-6 | 0.40 | 0.40 | 392 | 329 | 1130 | **33** |
| 1,3,5-Trimethylbenzene | 108-67-8 | 0.40 | 0.40 | 392 | 329 | 1130 | **33** |
| Benzene | 71-43-2 | 0.30 | 1.00 | 6359 | 3596 | 7177 | **360** |
| Styrene | 100-42-5 | 0.30 | 1.00 | 1448 | 1018 | 2736 | **102** |
| diisopropylbenzene | 99-87-6 | 0.40 | 0.40 | 216 | 198 | 764 | **20** |
| Toluene | 108-88-3 | 0.30 | 1.00 | 2567 | 1656 | 3960 | **166** |
| m-/p-Xylene | 106-42-3 | 0.30 | 1.00 | 1012 | 745 | 2134 | **74** |
| ortho-xylene | 95-47-6 | 0.30 | 1.00 | 1012 | 745 | 2134 | **74** |
| tert-Butylbenzene | 98-06-6 | 0.40 | 0.40 | 259 | 232 | 871 | **23** |
| Ethylbenzene | 100-41-4 | 0.30 | 1.00 | 1130 | 820 | 2308 | **82** |
| Cumene | 98-82-8 | 0.40 | 0.40 | 565 | 453 | 1463 | **45** |
| n-propylbenzene | 103-65-1 | 0.40 | 0.40 | 489 | 399 | 1321 | **40** |
| n-butylbenzene | 104-51-8 | 0.40 | 0.40 | 209 | 192 | 746 | **19** |
| sec-Butylbenzene | 135-98-8 | 0.40 | 0.40 | 241 | 218 | 826 | **22** |
| naphthalene | 91-20-3 | 0.50 | 1.00 | 1040 | 782 | 2300 | **78** |
